# Transcriptomic insights into developmental arrest in fluorescent labeling transgenic Asian elephant (*Elephas maximus*) embryos via inter-order cloning

**DOI:** 10.3389/fcell.2025.1532962

**Published:** 2025-02-20

**Authors:** Peachanika Pankammoon, Yubo Qing, Heng Zhao, Deling Jiao, Honghui Li, Fengchong Wang, Thanapa Wiriyahdamrong, Jianxiong Guo, Wengui Li, Phongsakorn Chuammitri, Chatchote Thitaram, Hongjiang Wei, Anucha Sathanowongs

**Affiliations:** ^1^ Faculty of Veterinary Medicine, Chiang Mai University, Chiang Mai, Thailand; ^2^ Science and Technology Department of Yunnan Province, Yunnan Province Key Laboratory for Porcine Gene Editing and Xenotransplantation, Kunming, China; ^3^ College of Veterinary Medicine, Yunnan Agricultural University, Kunming, China; ^4^ College of Animal Science, Yunnan Agricultural University, Kunming, China

**Keywords:** Asian elephant (*Elephas maximus*), embryonic arrest, interspecies somatic cell nuclear transfer technique (iSCNT), interspecies cloning, RNA sequencing (RNAseq), transcriptomic analysis

## Abstract

**Introduction:**

Asian elephants (*Elephas maximus*) provide a unique model for studying cloning in large mammals. As an endangered species with declining populations and limited oocyte availability, interspecies somatic cell nuclear transfer (iSCNT) combined with transcriptomic analysis holds promise for advancing iSCNT embryonic arrest development and further facilitating applications in conservation efforts, therapeutic cloning, and regenerative medicine.

**Methods:**

This study conducted low-input RNA sequencing analyses on transgenic Asian elephant-pig (AE-P) inter-order cloned embryos expressing enhanced green fluorescent protein (EGFP) at the 2- and 4-cell stages. Differential gene expressions, pathway enrichment, and hub gene analyses were performed to identify the molecular mechanisms and core genes influencing normal and arrest development.

**Results and Discussion:**

Approximately 25% of clean reads successfully aligned with the Asian elephant genome. The transcriptomic analysis revealed that inter-order cloned embryos with earlier cleavage at the 2- and 4-cell stages exhibited signs of residual transcriptomic memory and incomplete epigenetic reprogramming, while arrested embryos showed indications of nucleocytoplasmic incompatibility and nDNA-mtDNA mismatch. Hub gene analyses indicated core genes such as *NDUFC2*, *NDUFS3*, *NDUFAB1*, *SDHC*, *SDHB*, *NUP54*, *NUP43*, *NUP37*, *NDC1*, *CDK1*, and *CCNB1* linked to energy production, nucleocytoplasmic transport, and cell cycle regulation highlighting the overall challenges in cloning Asian elephant inter-order embryos. Altogether, the analysis of high-throughput sequencing enhances the reliability of iSCNT production in this study, advancing our understanding of cellular reprogramming and molecular roadblocks in AE-P inter-order cloned embryos. Transcriptomic analyses have identified key factors contributing to developmental barriers in iSCNT, offering valuable insights into the complexities of these challenges.

## 1 Introduction

The Asian elephant (*Elephas maximus*) is classified as an Endangered species by the International Union for Conservation of Nature because of a declining population across multiple countries (Songer et al., 2016; Liu et al., 2017; Fernando et al., 2019). Research on assisted reproductive technologies (ART) for this species faces challenges surrounding the lack of publications, ethical concerns, and the collection of reproductive samples. Advanced ART techniques such as somatic cell nuclear transfer (SCNT) within the same species have shown potential (Campbell et al., 2007; Ogura et al., 2013) but remain impractical in Asian elephants due to the limited availability of sources of ovaries and oocytes. As a result of these limitations, interspecies SCNT (iSCNT) has emerged as an alternative tool, enabling the production of iSCNT embryos, including those of the Asian elephant (Sathanawongs et al., 2010; Techakumphu et al., 2010; Nguyen et al., 2022).

The iSCNT technique employs recipient cytoplasmic factors in mature (MII) enucleated oocytes to reprogram donor nuclei from different species (Jullien et al., 2011; Lagutina et al., 2013; Borges and Pereira, 2019). However, a high incidence of embryonic arrest occurs as the phylogenetic distance between the donor cell and recipient cytoplast increases, resulting in *in vitro* and *in vivo* (IVV) developmental failures (Lagutina et al., 2013; Son et al., 2021; Nguyen et al., 2022; Srirattana et al., 2022). For instance, interspecies cloned lycaon-dog fetuses exhibited mitochondrial DNA (mtDNA) heteroplasmy with the donor nuclear DNA (nDNA), which impaired pyruvate metabolism and reduced iSCNT survival (Son et al., 2021). Another meta-analysis of amino acid sequences uncovered that inter-order rhesus monkey-cow iSCNT embryos encountered nuclear DNA and mitochondrial DNA (nDNA-mtDNA) incompatibility, lowering the blastocyst rates (Kwon et al., 2016). Additionally, microarray analyses of Przewalski’s gazelle iSCNT embryos revealed irregular transcriptome reprogramming, marked by improper degradation of maternal transcripts, incomplete activation of transcription factors, abnormal gene expression and mitochondrial dysfunction (Zuo et al., 2014). Furthermore, our previous study on Asian elephant-pig (AE-P) inter-order embryos reported embryonic development both *in vitro* and *in vivo*, revealing significantly low cleavage and blastocyst, with developmental arrest observed in 61.92% of embryos at the 2-cell stage and 82.53% at the 4-cell stage, highlighting cross-species barriers (Nguyen et al., 2022). Factors affecting iSCNT embryonic development include aberrant epigenetic reprogramming, incomplete maternal-to-zygotic transition (MZT) and embryonic genome activation (EGA), as well as incompatibilities in combining genetic material from different species (Lagutina et al., 2010; Lagutina et al., 2013; Zuo et al., 2014; Halstead et al., 2020; Zhou et al., 2020). However, the biological and molecular mechanisms underlying the *in vitro* development of AE-P iSCNT embryos remain unexplored; therefore, this study aims to investigate the transcriptomic profiles and gene expression of these embryos.

Accurate identification of the donor genome is essential for understanding the molecular mechanisms in iSCNT-reconstructed embryos through transcriptome analysis. Our previous study on AE-P inter-order cloning employed megaplex PCR using species-specific primers to verify the presence of the donor genome (Nguyen et al., 2022). However, this approach remained challenging in the possibility of producing iSCNT embryos without containing the donor genome, due to failed iSCNT embryos retaining the recipient genome, and the need to sacrifice limited embryonic tissue for PCR confirming (Nguyen et al., 2022). To overcome these issues, donor cells expressing enhanced green fluorescent protein (EGFP) provide an effective alternative, serving as a visible marker for genome detection in preimplantation transgenic cloned embryos (Lu et al., 2018). Previous studies in a transgenic canine model compared the promoters for EGFP transfection in donor cells prior to SCNT processes. The results found that EGFP regulated by the CMV promoter was strongly detectable at both organismal and cellular levels, whereas the hEF1α promoter was silenced by DNA methylation during early embryogenesis (Eun et al., 2020). This approach allows high throughput sequencing analyses without compromising the limited embryonic tissue in iSCNT-derived embryos. This transcriptome analysis of embryos can be conducted using tools such as microarrays (Zuo et al., 2014) and next-generation sequencing (NGS) technologies like RNA sequencing (RNA-seq) (Zhou et al., 2020; Zhang W. et al., 2023) or single-cell RNA sequencing (scRNA-seq) (Liu et al., 2016; Halstead et al., 2020; He et al., 2022). NGS has been utilized to study gene expressions during the early development of human-arrested embryos produced from *in vitro* fertilization (IVF) (Zhang W. et al., 2023), intracytoplasmic sperm injection (He et al., 2022), and SCNT (Liu et al., 2016). This technology provides precise insights into the expression patterns of hub genes and transcriptional pathways involved in embryonic development, offering improved production efficiency and advances in iSCNT research (Wu et al., 2018).

We aim that fluorescent labeling could provide precious selection of transgenic iSCNT embryos for the transcriptomic analysis. We classified normal developmental stages (Dev group) at the timing post activation, 24 h for 2-cell stage and 48 h for 4-cell stage, compared to those assuming developmental arrest (Arr group), 48 h apart of each stage. We identify differentially expressed genes (DEGs), core genes, and signaling pathways crucial for their development. Our findings detail the biological and molecular mechanisms underlying these embryos and support the application of iSCNT technology in this large mammalian model for further applications in animal therapeutic cloning and related areas within regenerative medicine.

## 2 Materials and methods

All chemical reagents used in this study were purchased from Sigma-Aldrich (St. Louis, MO, United States) unless otherwise stated.

### 2.1 EGFP Asian elephant donor cell preparation

The procedure for isolating Asian elephant fibroblasts (AEF) was performed as described in our previous reports (Nguyen et al., 2022). In brief, somatic cells were isolated from the ear skin tissues of a 6-year-old female Asian elephant. The primary cells were cultured in tissue culture media containing Dulbecco’s minimum essential medium (DMEM) supplemented with 10% FBS at 37°C in a 5% CO_2_ humidified atmosphere.

**FIGURE 1:**
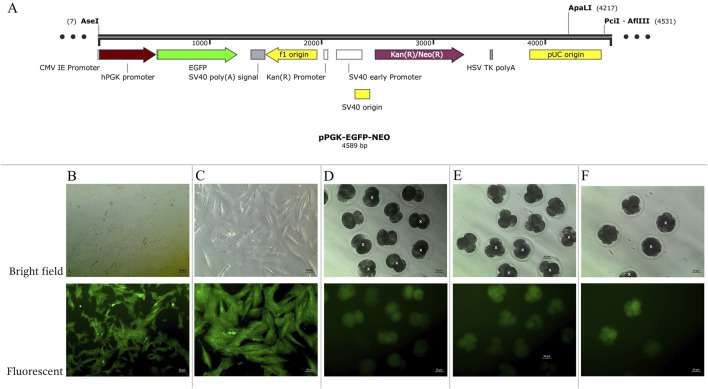
**(A)** Structure of pPGK-EGFP-NEO linearized plasmids. **(B–F)** Enhance green fluorescent protein (EGFP) expression examined under a fluorescent microscope in bright and fluorescent fields. **(B, C)** transgenic Asian elephant fibroblasts **(D)** Asian elephant-pig inter- order cloned embryos at the 2-cell stage **(E)** at the 4-cell stage **(F)** at the 8-cell stage. White “X” indicated negative expression EGFP embryos.

To generate AEFs expressing EGFP, the pPGK-EGFP-NEO vector was constructed and used for transfection (Figure 1A). This vector contains the CMV, I.E., promoter to drive EGFP expression and a neomycin resistance (NEO) cassette for selecting transfected cells. The plasmid DNA was linearized using the ApaI restriction enzyme (Thermo Fisher Scientific, United States). The cultivated AEFs at 80% confluence were transfected with 30 µg of linearized plasmid DNA using Lipofectamine-LTX (Invitrogen, CA, United States) according to the manufacturer’s instructions, and then cryopreserved in liquid nitrogen until use. EGFP-expressing AEFs were subcultured until they reached 80% confluence at passages 4-9 and utilized as nuclear donors for inter-order AE-P iSCNT.

### 2.2 Porcine recipient oocytes collection and *in vitro* maturation

Porcine ovaries were obtained from local slaughterhouses and kept in Phosphate Buffer Saline at 25°C–37°C until use. The cumulus-oocyte complexes (COCs) were collected as previously described (Wei et al., 2013). Porcine COCs with homogeneous cytoplasm and multiple layers of cumulus cells were selected for *in vitro* maturation (IVM). Approximately 50 COCs were cultured in 200 µL of IVM medium (TCM-199 medium supplemented with 0.1 mg/mL pyruvic acid, 0.1 mg/mL L-cysteine hydrochloride monohydrate, 10 ng/mL epidermal growth factor, 10% (v/v) porcine follicular fluid, and 10 IU/mL eCG and hCG (Teikoku Zouki Co., Tokyo, Japan)), covered with mineral oil, and incubated at 38.5°C in an atmosphere of 5% CO2. After 42–44 h (h) of IVM, the mature COCs were denuded by gentle pipetting in 1% Hyaluronidase enzyme to remove cumulus cells. Porcine mature oocytes at metaphase II (MII) with the presence of the first polar body were subsequently used as recipient cytoplasts for iSCNT.

### 2.3 Transgenic Asian elephant-pig iSCNT embryos and *in vitro* culture

The inter-order AE-P iSCNT protocol was adapted from our previous method (Wei et al., 2013; Nguyen et al., 2022). In brief, porcine MII oocytes were incubated in 0.1% µg/mL Demecolcine for 30–60 min (min) prior to enucleation. Enucleation was performed by aspirating the first polar body and the adjacent cytoplasmic protrusion using a micromanipulator. A single round-shaped EGFP-expressing AEF with a smooth margin was then injected into the perivitelline space of the enucleated recipient cytoplast. The reconstructed donor cell-recipient cytoplast complexes were fused using an embryonic cell fusion system in a fusion medium (0.25 M D-sorbic alcohol, 0.05 mM Mg(C_2_H_3_O_2_)_2_, 20 mg/mL BSA, and 0.5 mM HEPES) with a single direct current pulse of 250 V/mm for 25 µs (LF 201, Nepa Gene Co. Ltd., Tokyo, Japan). Following fusion, the transgenic AE-P iSCNT reconstructed embryos underwent delayed activation for 1–2 h in a porcine zygote medium-3 (PZM-3) medium, followed by activation with a single direct current pulse of 150 V/mm for 100 µs (LF 201, Nepa Gene Co. Ltd., Tokyo, Japan), in an activation medium (0.25 M D-sorbic alcohol, 0.01 mM Ca(C_2_H_3_O_2_)_2_, 0.05 mM Mg(C_2_H_3_O_2_)_2_, and 0.1 mg/mL BSA). Post-activation, the reconstructed embryos were cultured in the PZM-3 medium supplemented with 5 μg/mL CB for 2 h at 38.5°C in 5% CO_2_. Finally, the embryos were washed three times and cultured in fresh PZM-3 at 38.5°C in a humidified atmosphere of 5% CO_2_, 5% O_2_, and 90% N_2_ (APM-30D, ASTEC, Japan).

Transgenic AE-P cloned embryos were categorized into two groups: developmental (Dev) and arrested (Arr), based on porcine developmental stages and timings. The Arr embryos, which showed no signs of degeneration, were evaluated 48 h after their corresponding Dev groups at the same embryonic stage. Specifically, the focus was on the 2-cell (Dev2cell at 24-h post-activation and Arr2cell at 72 h post-activation) and 4-cell stages (Dev4cell at 48-h post-activation and Arr4cell at 96-h post-activation). GFP expressions in these transgenic embryos were examined under an inverted fluorescence microscope for genomic identification and subsequent RNA-seq analysis.

### 2.4 Molecular identification of Asian elephant donor genome

EGFP-positive embryos were selected for donor genome screening via PCR, utilizing two sets of primers: species-specific primers targeting the Asian elephant *TYR* gene and *GFP* primers (Supplementary Table S1), ensuring an accurate Asian elephant genome for further RNA-seq analysis. Transgenic embryos at the 2-cell (Dev2cell and Arr2cell), 4-cell (Dev4cell and Arr4cell), and 8-cell (Dev8cell at 72-h post-activation) stages were chosen for genomic DNA extraction. EGFP-expressing AEFs were used as a positive control (PC) group. The selected embryos were washed three times in PBS and individually transferred into 3 µL of DNA extraction lysis buffer. Genomic DNA was extracted using a cell lysis reaction at 65°C for 30 min, then 95°C for 10 min using the Proflex PCR system (Thermo Fisher Scientific, MA, United States). Each genomic DNA template was divided into two groups for amplification with two different primers. The 25 µL PCR reaction volume contained 1.5 µL of DNA template, 1 µL of forward and reverse primer respectively, 12.5 µL of PrimeScript Buffer, 1 µL of Oligo dT Primer, and 0.5 µL of DNA polymerase (Takara, Tokyo, Japan). The PCR thermal cycling conditions were as follows: initial denaturation for 5 min at 94°C; 10 cycles of 98°C for 15 s, 68°C for 25 s, and 72°C for 40 s; 25 cycles of 98°C for 15 s, 58°C for 25 s, and 72°C for 40 s; followed by a final extension at 72°C for 5 min. The PCR amplicons were then electrophoresed on 1% agarose gel electrophoresis.

### 2.5 Sample collection for library construction and RNA-Sequencing

In this study, transgenic AE-P iSCNT embryo groups included Dev2cell, Arr2cell, Dev4cell, Arr4cell, Dev8cell, and Dev16cell (144-h post-activation). Each group consisted of pooled samples from approximately 10–20 EGFP-expressing iSCNT embryos, with three biological replicates per pool, except for Dev8cell and Dev16cell due to limited sample availability. The primary focus of this study was on the Dev2cell, Arr2cell, Dev4cell, and Arr4cell groups. To prepare the embryos, the zona pellucida of the selected positive EGFP embryos was removed by incubation in 1 mg/mL of Pronase for 2 min, followed by three washes in DPBS. The embryos were then placed in cell lysis buffer and stored at −80°C prior to SMART-Seq2 amplification and library construction. Library preparation and RNA-seq were conducted by Beijing Genomics Institute (Beijing, China). RNA-seq was performed on the MGISEQ-2000 platform. Clean data were received after filtering out raw data contaminated with adapters, unknown base N content, and low-quality reads using SOAPnuke (v1.6.5). Data quality was assessed, including statistics for Q20, Q30, and GC content. The clean reads were aligned to the Asian elephant reference genome (GCF_024166365.1) using HISAT2 (v2.2.1) and aligned to the reference gene sequence with Bowtie2 (v2.4.5) software. The Fragments Per Kilobase of transcript per Million Mapped reads (FPKM) values of each gene were calculated using RSEM (v1.3.1) based on gene length and the number of reads mapped to estimate gene expression levels. Differential expression analysis was performed using DESeq2. Genes with a false discovery rate (FDR, Q value) < 0.05 and a log2 fold change ≥1 were identified as differentially expressed.

### 2.6 Transcriptome analysis and protein-protein interactions

Transcriptome analyses were performed and visualized using the Dr.Tom I web-based platform (BGI, Beijing, China). DEGs were classified into upregulated and downregulated genes for enrichment and pathway analysis. According to the DEGs results, hierarchical clustering analysis was performed on the union differential genes using the pheatmap function in RStudio. Gene Ontology (GO) and Kyoto Encyclopedia of Genes and Genomes (KEGG) enrichment analyses were on DEGs using the phyper function in RStudio. Finally, P-values were calculated and adjusted using the FDR method.

Hub genes in the Dev2cell, Dev4cell, Arr2cell, and Arr4cell embryos were identified through the analysis of enriched DEGs selected for the network construction of protein-protein interactions (PPI). The PPI network was established using Cytoscape (v3.10.2) software, based on data obtained from the STRING database. The visualization of network relationships was analyzed through the CytoHubba plugin in Cytoscape, including the maximal clique centrality (MCC) and degree centrality (Chin et al., 2014). Significant hub genes were identified by the intersection of two algorithms and illustrated using Venn diagrams. Subsequently, KEGG analysis was used once more to analyze the enrichment pathways of the hub genes (Q < 0.05).

## 3 Results

### 3.1 Molecular identification of the Asian elephant donor genome

**FIGURE 2:**
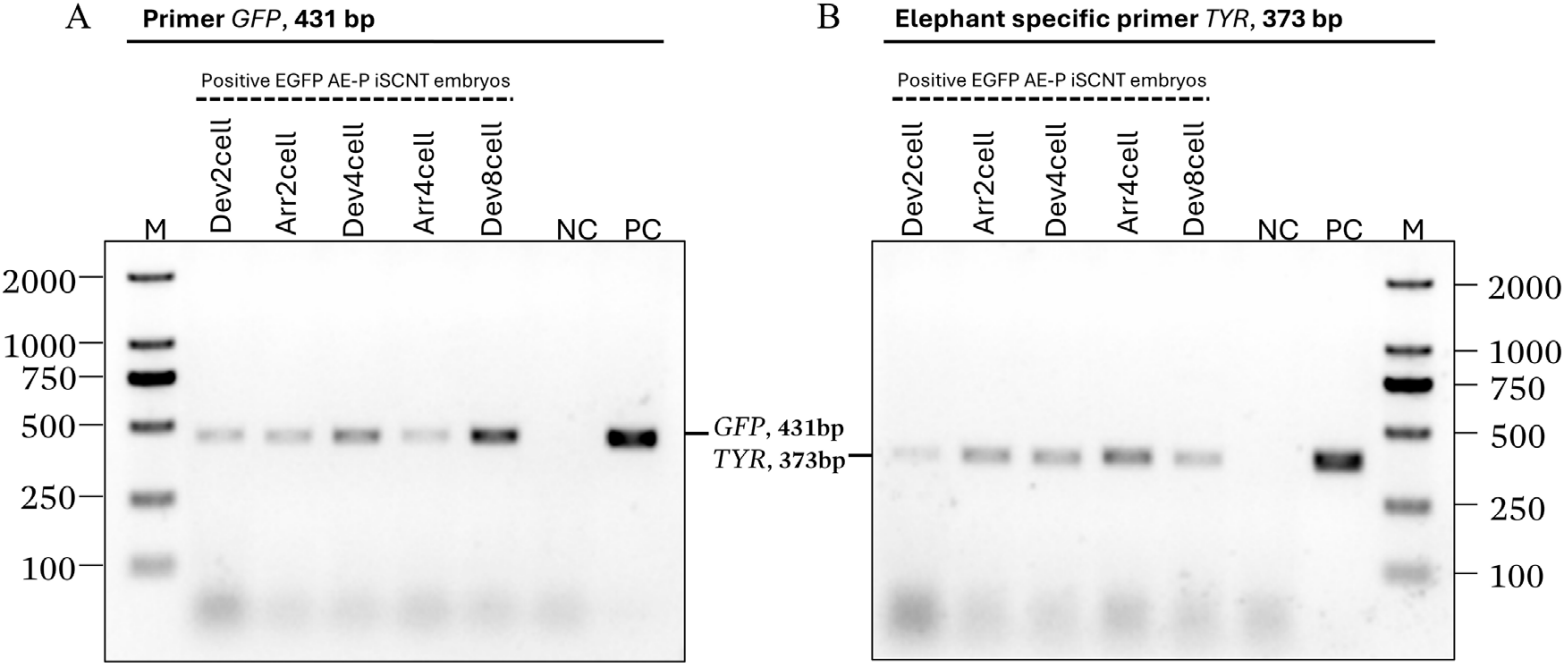
Asian elephant genome identification of transgenic Asian elephant-pig inter-order cloned embryos expressing EGFP at the 2-cell, 4-cell, and 8-cell stage. **(A)** The primers of the *GFP* gene (431 bp). **(B)** Asian elephant-specific primers *TYR* gene (373 bp) (M, Marker 2,000 bp; NC, negative control; PC, positive control; Dev, timely developmental group, Arr, arrested developmental group).

Transgenic AE-P iSCNT embryos and AEF expressing EGFP were examined under a fluorescent microscope (Figures 1B–F), and only embryos expressing EGFP were selected for donor genome screening. EGFP-positive AEF cultures served as positive controls. Genomic DNA from individual transgenic AE-P iSCNT embryos was divided into two groups for amplification using two different primers. The molecular analysis confirmed the presence of the Asian elephant genome in EGFP-positive embryos at the 2-cell (Dev2cell and Arr2cell), 4-cell (Dev4cell and Arr4cell), and 8-cell (Dev8cell) stages, as indicated by the presence of both *GFP* and *TYR* genes (Figure 2). This finding highlights the effectiveness of EGFP-tagged somatic cells in marking embryos for sequencing analysis, even with limited embryonic material.

### 3.2 RNA sequencing overview of transgenic Asian elephant inter-order embryos

RNA sequencing of transgenic AE-P iSCNT embryos was conducted using the MGISEQ-2000 platform, yielding 464 million raw reads from fourteen groups of Dev and Arr embryos. After data filtering, an average of 25.96 million clean reads per sample was generated, totaling approximately 5.19 Gb of data per sample. The quality of the reads was high, with an average Q20 of 98.09%, Q30 of 93.10%, and a GC content of 48.86% (Supplementary Table S2). On average, 24.95% of the clean reads aligned to the Asian elephant genome, while 15.12% aligned to the gene set (Supplementary Table S3). In total, 19,581 genes were detected, including 19,446 known genes, 135 newly predicted genes, and 12,728 new transcripts. This comprehensive RNA sequencing analysis provides insight into the transcriptomic landscape of transgenic AE-P inter-order cloned embryos, encompassing differential gene expression, GO and KEGG enrichment analyses, and the identification of key hub genes through PPI network analysis.

### 3.3 Differential gene expression and enrichment analysis

RNA-seq analysis revealed gene expression dynamics with total DEGs analyzed for each comparison. DEGs were identified as upregulated and downregulated, visualized through volcano plot, using FDR of Q < 0.05 and a log2 fold change ≥1. Heatmap clustering of DEGs was generated using the log2(FPKM+1) values of DEGs for visualization. Enrichment analysis GO terms and KEGG pathways with Q < 0.05 were considered significantly enriched by DEGs.

#### 3.3.1 Timely developmental group comparisons

**FIGURE 3:**
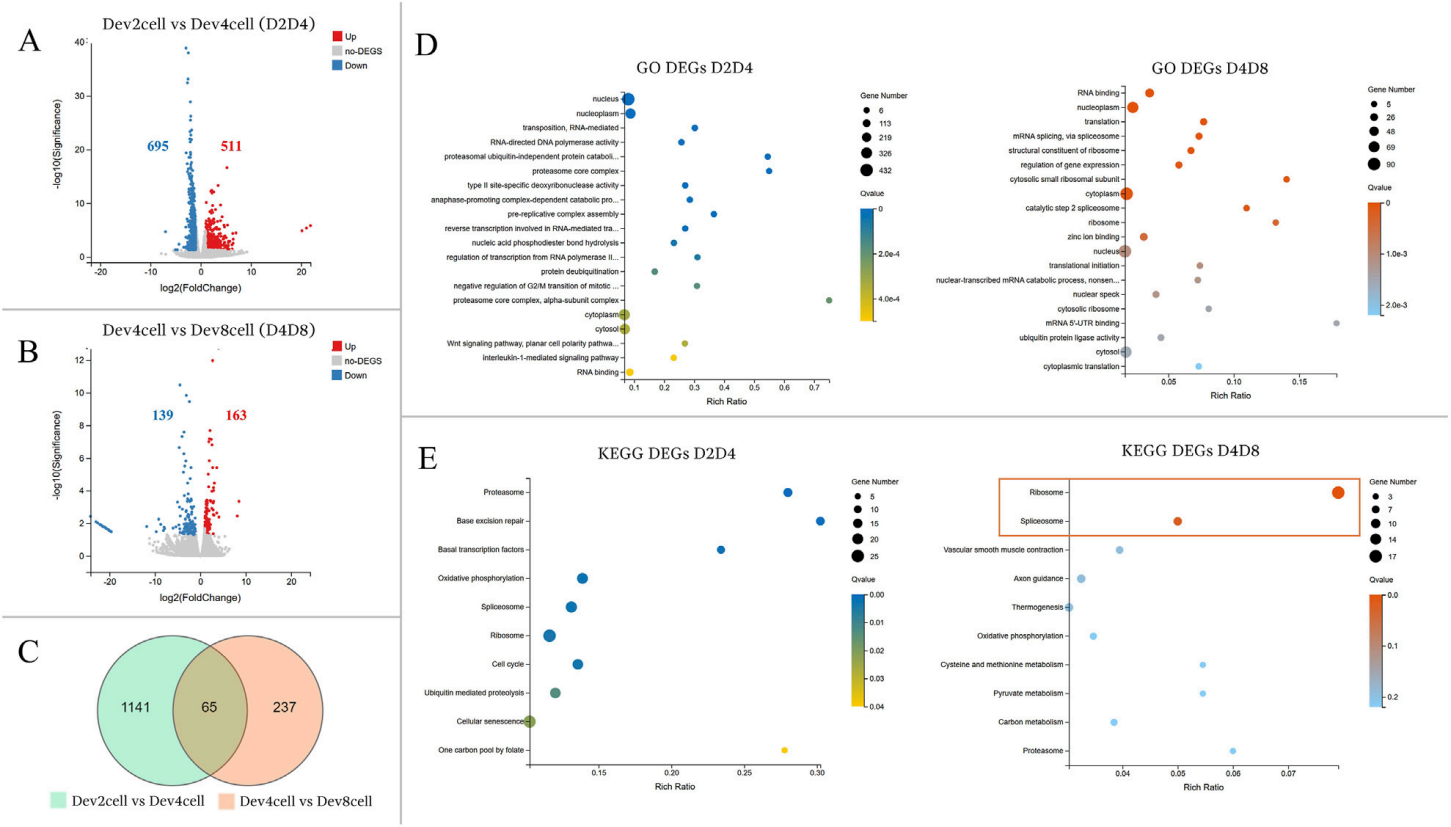
**(A, B)** Volcano plots illustrating differential gene expression analyses between the Dev and Arr groups. The Y-axis represents the -log10 of the adjusted p-value (Q value), while the X-axis shows the log2 fold change (log2FC ≥ 1, Q < 0.05). **(A)** Volcano plot showing DEGs between Dev2cell and Dev4cell (D2D4) embryos. **(B)** Volcano plot displaying DEGs between Dev4cell and Dev8cell (D4D8) embryos. Red dots indicate significantly upregulated genes, while blue dots indicate significantly downregulated genes and grey dots represent non- significant DEGs. **(C)** Venn diagram showing overlapping DEGs between the D2D4 and D4D8 groups, highlighting the total number of DEGs and their intersections. **(D, E)** Enrichment analyses for total DEGs from the D2D4 and D4D8 groups. The X-axis shows the gene ratio percentage, and the Y-axis represents GO terms or KEGG pathways. Circle size corresponds to the gene count, and color intensity reflects significance (Q < 0.05). **(D)** GO enrichment bubble chart highlighting the top 20 GO terms with the lowest Q values. **(E)** The KEGG pathway enrichment bubble chart shows the top 10 pathways, with significantly enriched pathways in the D4D8 group indicated by a square.

The transcriptional profiles were analyzed between the developmental stages of Dev2cell vs Dev4cell (D2D4) and Dev4cell vs Dev8cell (D4D8) (Figure 3). The D2D4 comparison identified 1,206 DEGs, of which 511 were upregulated and 695 were downregulated (Figure 3A). In the D4D8 comparison, 302 DEGs were found, with 163 upregulated and 139 downregulated (Figure 3B). A Venn diagram illustrated the overlap DEGs between these comparisons, showing 1,141 DEGs in D2D4, 237 DEGs in D4D8, and 65 DEGs intersecting between both groups (Figure 3C). We performed downstream enrichment analyses on the overlapping DEGs identified in the Venn diagram to investigate the molecular transitions between these stages. In the D2D4 group, significant enrichment was observed in GO processes such as nucleus, nucleoplasm, RNA mediated transposition, RNA-directed DNA polymerase activity, and proteasome ubiquitin activity, highlighting their roles in DNA replication, transcription regulation, cell cycle control, and protein degradation. In contrast, the D4D8 group showed enriched terms related to RNA binding, nucleoplasm, translation, and mRNA splicing, indicating a shift toward translation and mRNA processing (Figure 3D; Supplementary Table S4). KEGG pathway analysis for the D2D4 group revealed enrichment in pathways such as proteasome, base excision repair, basal transcription factors, oxidative phosphorylation, and spliceosome pathways (Figure 3D). KEGG enrichment for the D4D8 group displayed significant involvement in two pathways including ribosome and spliceosome pathways (Figure 3E; Supplementary Table S5). Overall, the DEGs between the 2- and 4-cell stages were associated with gene activation and protein degradation regulation, while DEGs between 4- and 8-cell indicated a shift toward translation-related activities.

#### 3.3.2 Timely and arrested developmental groups comparisons

**FIGURE 4:**
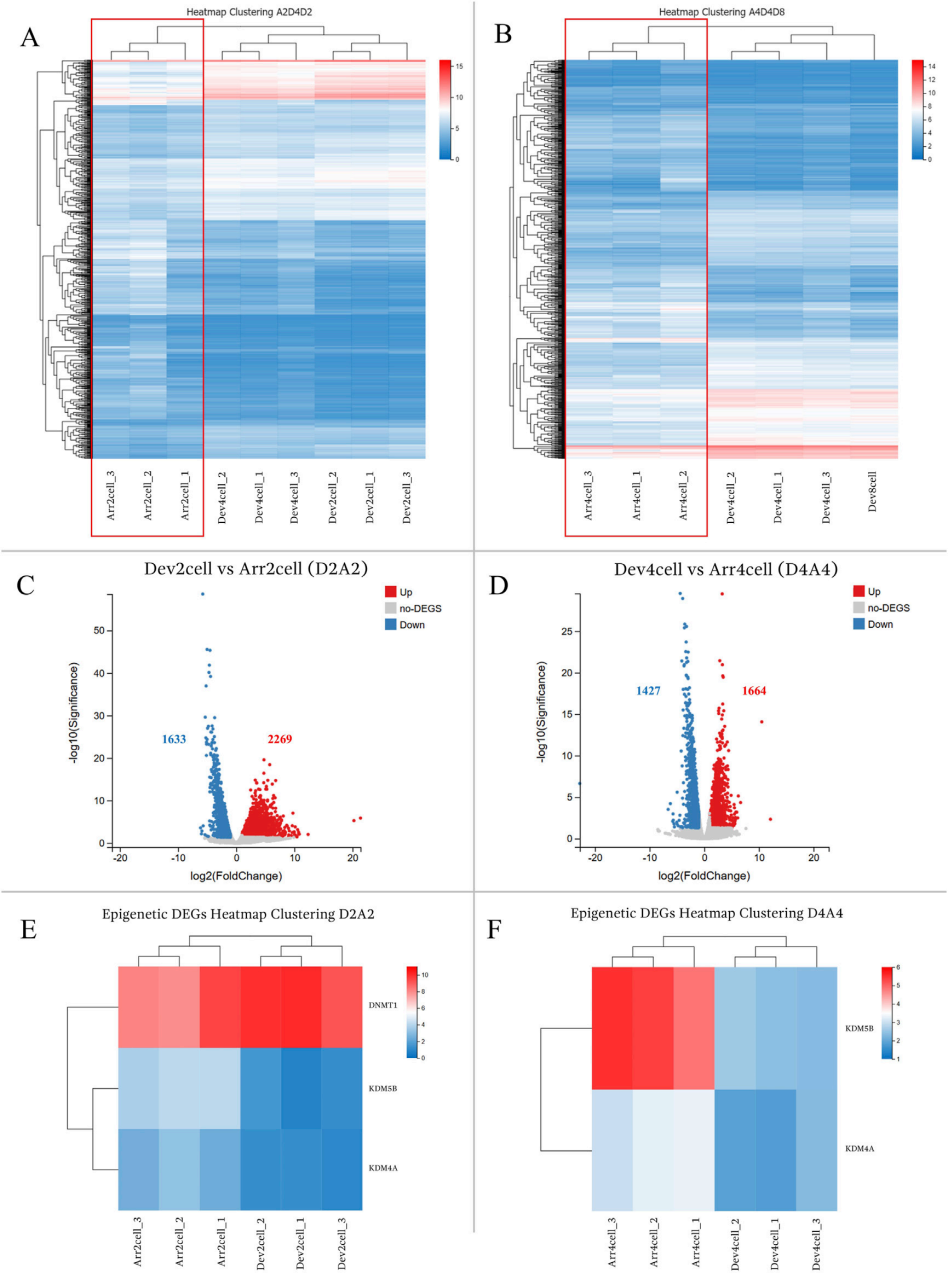
**(A,B)** Heatmap clustering illustrating gene expression patterns in transgenic Asian elephant-pig inter-order cloned embryos between Dev and Arr groups across the 2-cell to 8-cell stages. **(A)** Heatmap comparing Arr2cell, Dev2cell, and Dev4cell. **(B)** Heatmap comparing Arr4cell, Dev4cell, and Dev8cell. **(C,D)** Volcano plots displaying DEGs between Dev and Arr groups, with red dots for significantly upregulated genes, blue dots for significantly downregulated genes, and grey dots for non-significant DEGs (log2FC≥1,Q<0.05). **(C)** Volcano plot showing DEGs between Dev2cell and Arr2cell (D2A2). **(D)** Volcano plot showing DEGs between Dev4cell and Arr4cell (D4A4). **(E)** Heatmap showing significant DEGs related to epigenetics between D2A2. **(F)** Heatmap showing significant DEGs related to epigenetics between D4A4.

The comparison between the timely developmental and arrested groups exhibited significant gene expression differences. Heatmap clustering of global gene expression showed that Dev groups clustered closely within their respective groups, whereas Arr embryos (Arr2cell and Arr4cell) formed distinct clusters (Figures 4A, B; Supplementary Table S6). Differential gene expression analysis identified 3,902 DEGs in Dev2cell vs Arr2cell (D2A2), with 2,269 genes upregulated and 1,633 genes downregulated (Figure 4C). In the Dev4cell vs Arr4cell (D4A4) comparison, 3,091 DEGs were found, with 1,664 upregulated and 1,427 downregulated (Figure 4D). Following global gene expression heatmap clustering, we further analyzed epigenetic-related DEGs between D2A2 and D4A4 groups. The important epigenetic-related genes, including *DNMT1*, *DNMT3A*, *DNMT3B*, *TET1*, *TET2*, *TET3*, *KDM4A*, *KDM4B*, *KDM5A*, and *KDM5B* (Du et al., 2021), were screened for significant DEGs based on log2(FPKM+1) values. Among these, *DNMT1*, *KDM4A*, and *KDM5B* were significant DEGs in D2A2, while *KDM4A* and *KDM5B* were significant in D4A4 (Supplementary Table S6). Specifically, *DNMT1* was upregulated in D2 compared to A2 (Figure 4E), whereas *KDM5B and KDM4A* were upregulated in both A2 and A4 compared to D2 and D4 (Figure 4F).

**FIGURE 5:**
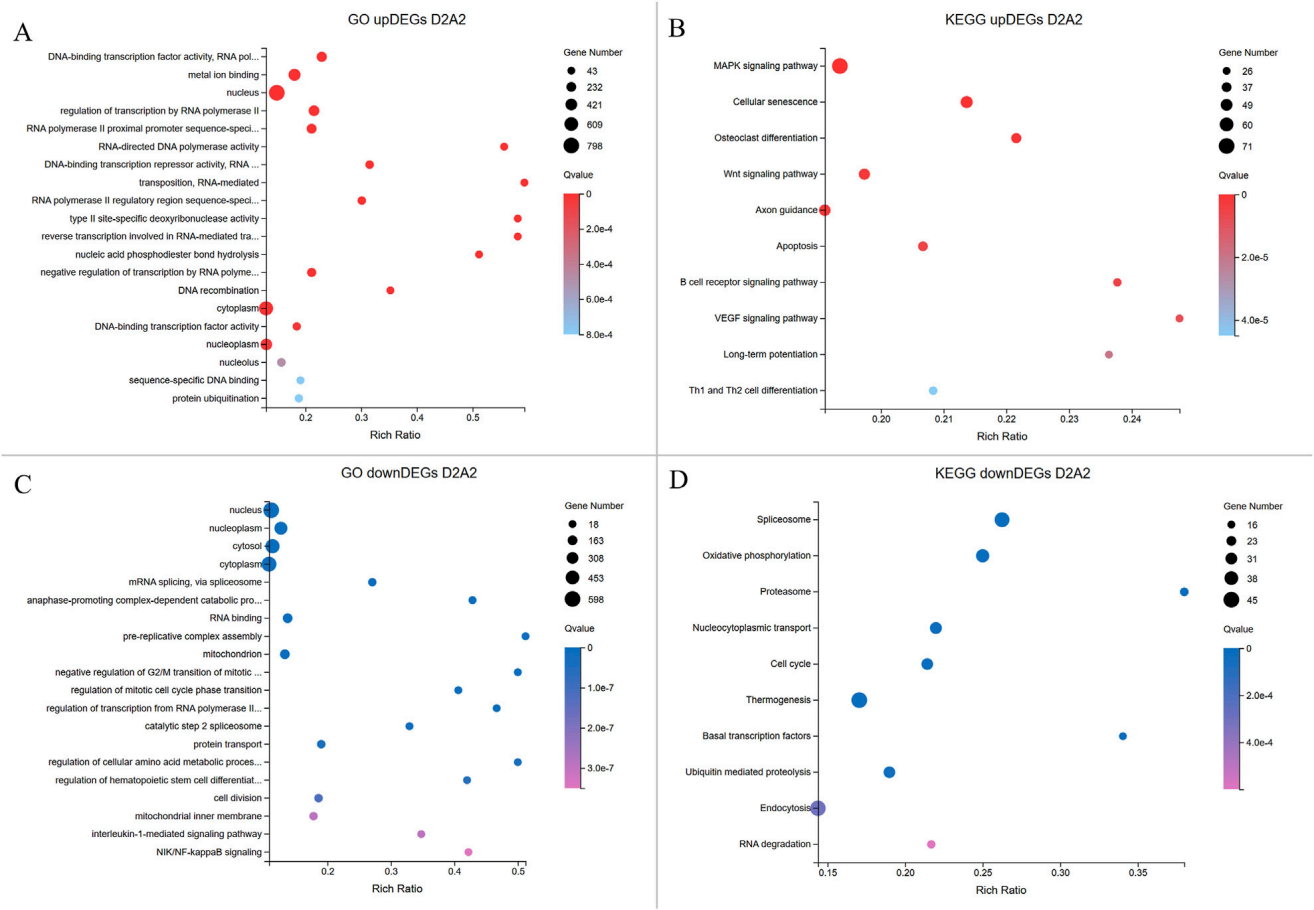
Enrichment analyses of upregulated and downregulated DEGs from the D2A2 group. The X-axis shows the gene ratio percentage, and the Y-axis represents the GO terms or KEGG pathways. The circle size reflects the gene count, while the color gradient indicates significance (Q < 0.05). **(A, B)** GO and KEGG pathway enrichment analyses for upregulated DEGs in Dev2cell. **(C, D)** GO and KEGG enrichment pathway analyses for upregulated DEGs in Arr2cell.

**FIGURE 6:**
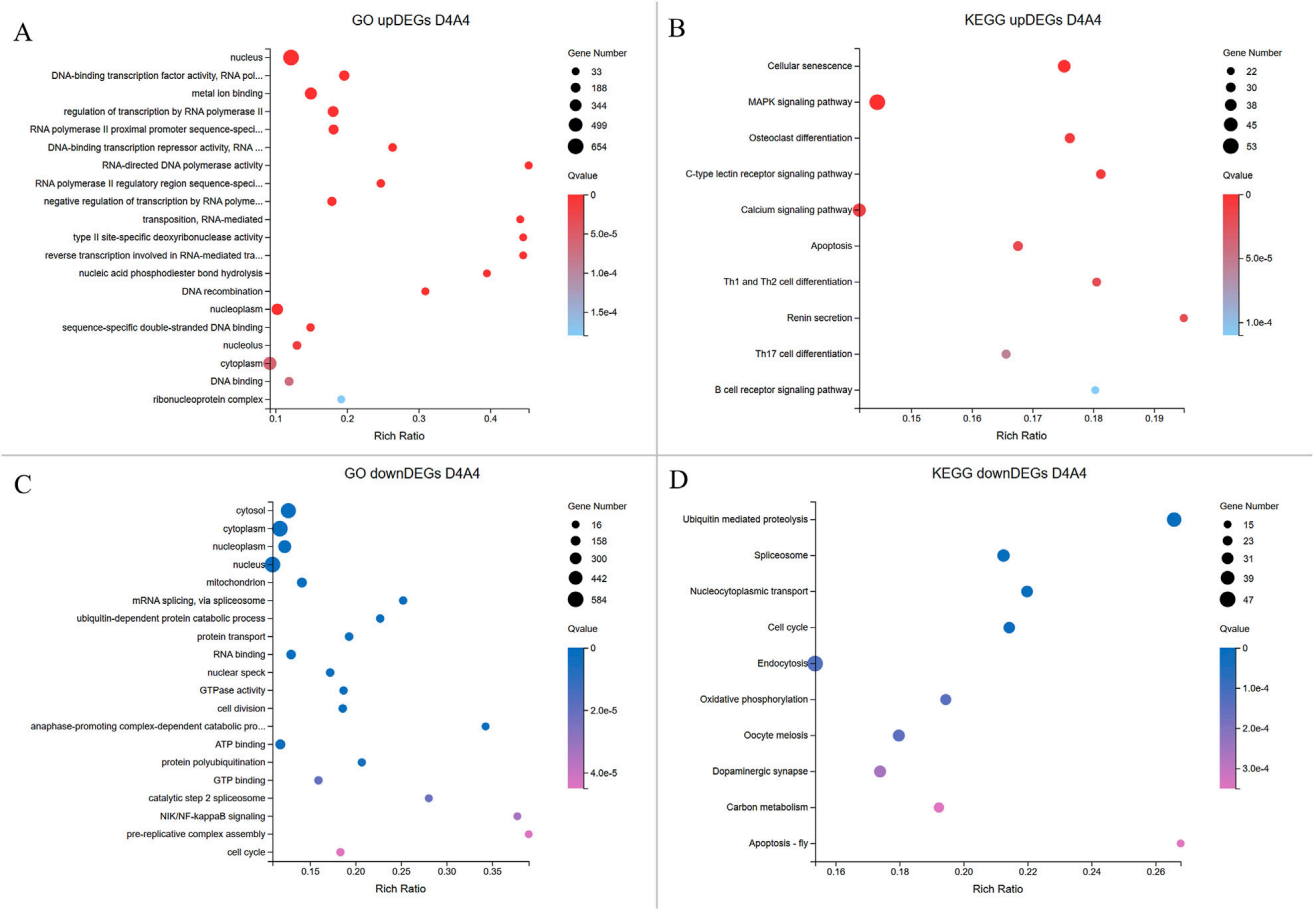
Enrichment analyses of upregulated and downregulated DEGs from the D4A4 group. The X-axis indicates the gene ratio percentage, while the Y-axis represents the GO terms or KEGG pathways. The circle size reflects the gene count, and the color gradient indicates significance (Q < 0.05). **(A, B)** GO and KEGG pathway enrichment analyses for upregulated DEGs in Dev4cell. **(C, D)** GO and KEGG pathway enrichment analyses for upregulated DEGs in Arr4cell.

Next, GO and KEGG enrichment analyses were conducted to explore the molecular mechanisms involved in transgenic AE-P embryo development based on the upregulated and downregulated DEGs in D2A2 and D4A4 embryos (Figures 5, 6). The enriched GO terms covered biological processes (BP), molecular functions (MF), and cellular components (CC) (Supplementary Table S4), while KEGG analysis revealed significant pathways during the early embryonic development (Supplementary Table S5).

In the D2A2 comparison, Dev2cell embryos showed 2,269 upregulated DEGs enriched in MF GO terms such as DNA-binding transcription factor activity, RNA polymerase II-specific, metal ion binding, RNA-directed DNA polymerase activity which play roles in transcription regulation, binding, and catalytic activity, while enrichments in CC and BP terms included nucleus, regulation of transcription by RNA polymerase II, RNA-mediated transposition (Figure 5A; Supplementary Table S4). In the KEGG analysis, MAPK and Wnt signaling pathways indicate active roles in cell proliferation, differentiation, and survival. Notably, other pathways were enriched in Dev2cell, such as osteoclast differentiation, cellular senescence, axon guidance, long-term potentiation, B cell receptor signaling, and Th1 and Th2 cell differentiation (Figure 5B; Supplementary Table S5). In contrast, Arr2cell embryos exhibited 1,633 upregulated DEGs enriched in several CC and BP terms such as nucleoplasm, cytosol, mRNA splicing, anaphase-promoting complex, pre-replicative complex assembly, mitochondrion, negative regulation of G2/M transition of mitotic cell cycle, regulation of mitotic cell cycle phase transition, protein turnover, and cell division. Additionally, BP terms related to stress response pathways including interleukin-1-mediated and NIK/NF-kappaB signaling suggested inflammatory or stress-related responses in these arrested embryos. MF terms indicated RNA binding (Figure 5C; Supplementary Table S4). KEGG analysis showed enriched pathways in spliceosome, oxidative phosphorylation, proteasome, nucleocytoplasmic transport and cell cycle (Figure 5D; Supplementary Table S5), pointing to disrupted RNA processing, energy metabolism, nuclear-cytoplasmic transport, and protein degradation roles in Arr2cell embryos.

In the D4A4 comparison, Dev4cell embryos showed 1,664 upregulated DEGs enriched in MF terms such as DNA-binding transcription factor activity, metal ion binding, RNA polymerase II-specific activity, DNA-binding transcription, and RNA-directed DNA polymerase activity, emphasizing roles in transcription regulation, binding, and catalytic activity. CC and BP terms were enriched in nucleus, regulation of transcription by RNA polymerase II, negative regulation of transcription by RNA polymerase II, and RNA-mediated transposition (Figure 6A; Supplementary Table S4). KEGG pathway analysis highlighted the MAPK signaling and unexpected pathways in the 4-cell stage AE-P iSCNT embryos, including osteoclast differentiation, C-type lectin receptor signaling, and various immune-mediated pathways (Figure 6B; Supplementary Table S5). In contrast, Arr4cell embryos exhibited 1,427 upregulated DEGs enriched in CC and BP terms such as mitochondrion, cytosol, nucleoplasm, mRNA splicing, ubiquitin-dependent protein catabolic process, and protein transport. MF terms included RNA binding, GTPase activity, ATP binding, and GTP binding, indicating binding and catalytic activity (Figure 6C; Supplementary Table S4). KEGG pathway analysis showed similarities to Arr2cell, including pathways such as ubiquitin-mediated proteolysis, spliceosome, nucleocytoplasmic transport, and cell cycle pathways, indicating roles in protein degradation, RNA splicing, nuclear-cytoplasm transport, and cell division in Arr4cell (Figure 6D; Supplementary Table S5).

### 3.4 Protein-protein interaction (PPI) network and enrichment analysis

**FIGURE 7:**
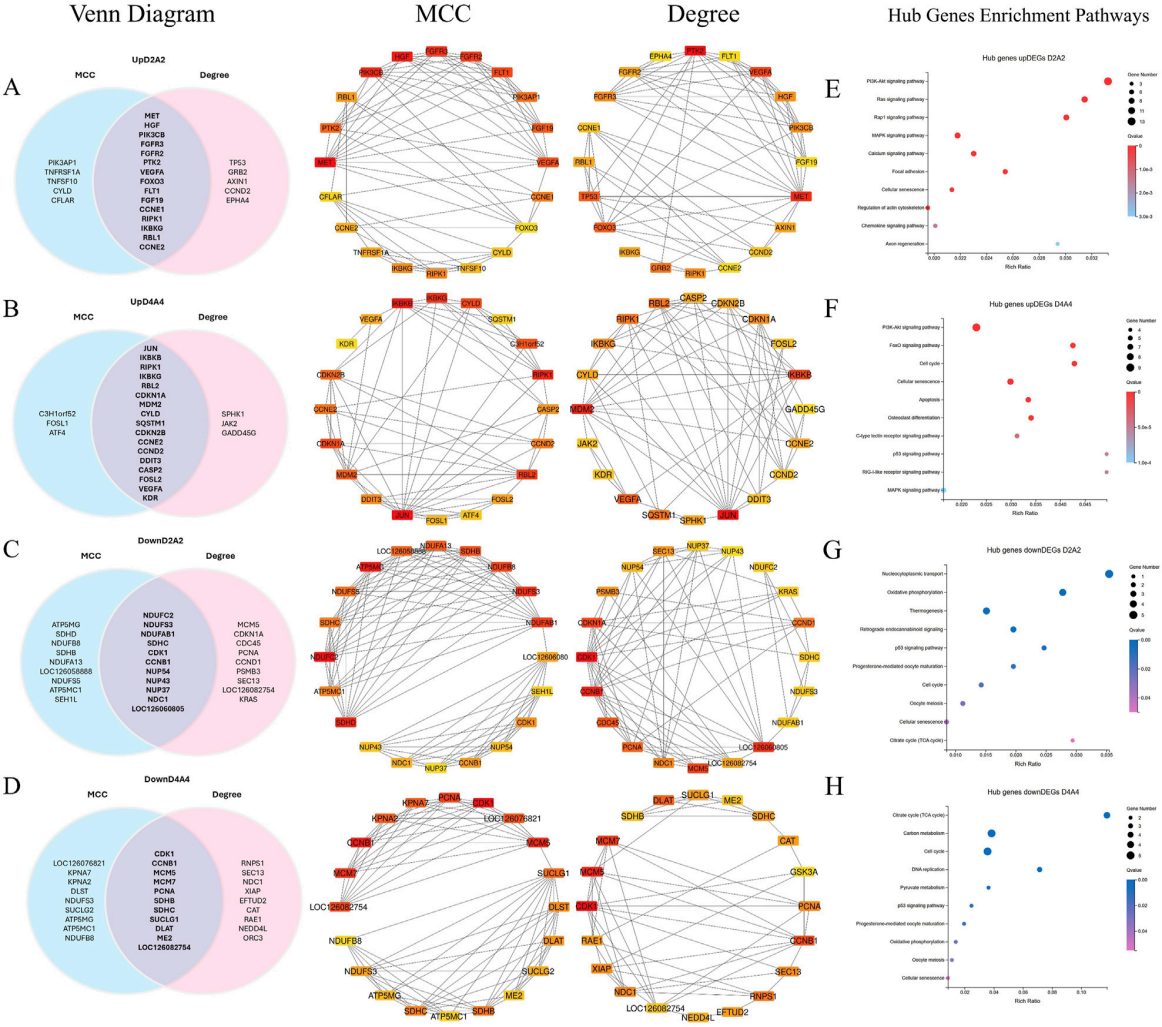
A PPI network was constructed, and hot module analysis was performed for upregulated DEGs in Dev2cell, Dev4cell, Arr2cell, and Arr4cell. The highest interaction score PPIs were selected to build the network. Genes with high interaction scores from MCC and Degree analyses are represented as squares, with protein interactions shown by connecting lines. Genes with higher interaction scores are highlighted in red, while those with lower scores are shown in yellow. Overlapping hub genes from the MCC and Degree algorithms were considered significant core genes and are represented in a Venn diagram. **(A)** Venn diagram illustrating the intersection of 15 core genes in the Dev2cell group. Two significant modules were identified from the PPI network using MCC and Degree algorithms. **(B)** Venn diagram showing 17 core genes in the Dev4cell group. **(C)** Venn diagram depicting 11 core genes in the Arr2cell group. **(D)** Venn diagram representing 11 core genes in the Arr4cell group. **(E–H)** Core genes were subsequently analyzed for enrichment pathways (Q < 0.05). **(E)** KEGG pathway enrichment associated with upregulated DEGs in Dev2cell. **(F)** KEGG pathway enrichment for upregulated DEGs in Dev4cell. **(G)** KEGG pathway enrichment for upregulated DEGs in Arr2cell. **(H)** KEGG pathway enrichment for upregulated DEGs in Arr4cell.

To identify the highly connected genes in the Dev2cell, Dev4cell, Arr2cell, and Arr4cell of AE-P iSCNT embryos, enriched DEGs were selected for PPI network analysis using Cytoscape software. The network relationships were conducted on nodes (genes) and edges (interactions) and analyzed through the CytoHubba plugin in Cytoscape, employing two algorithms of MCC and Degree centrality (Chin et al., 2014). Overlapping hub genes that were identified as significant across these two algorithms were considered core genes within the network (Figure 7). Genes consistently scoring high for connectivity were identified as core genes in D2A2 (Supplementary Table S7) and D4A4 (Supplementary Table S8). The biological functions and pathway enrichment related to these overlapping core genes were validated as significant (Q < 0.05) (Figure 7; Supplementary Table S9). The analysis underscored 15 core genes in the Dev2cell group, 17 in Dev4cell, and 11 each in Arr2cell and Arr4cell.

**FIGURE 8:**
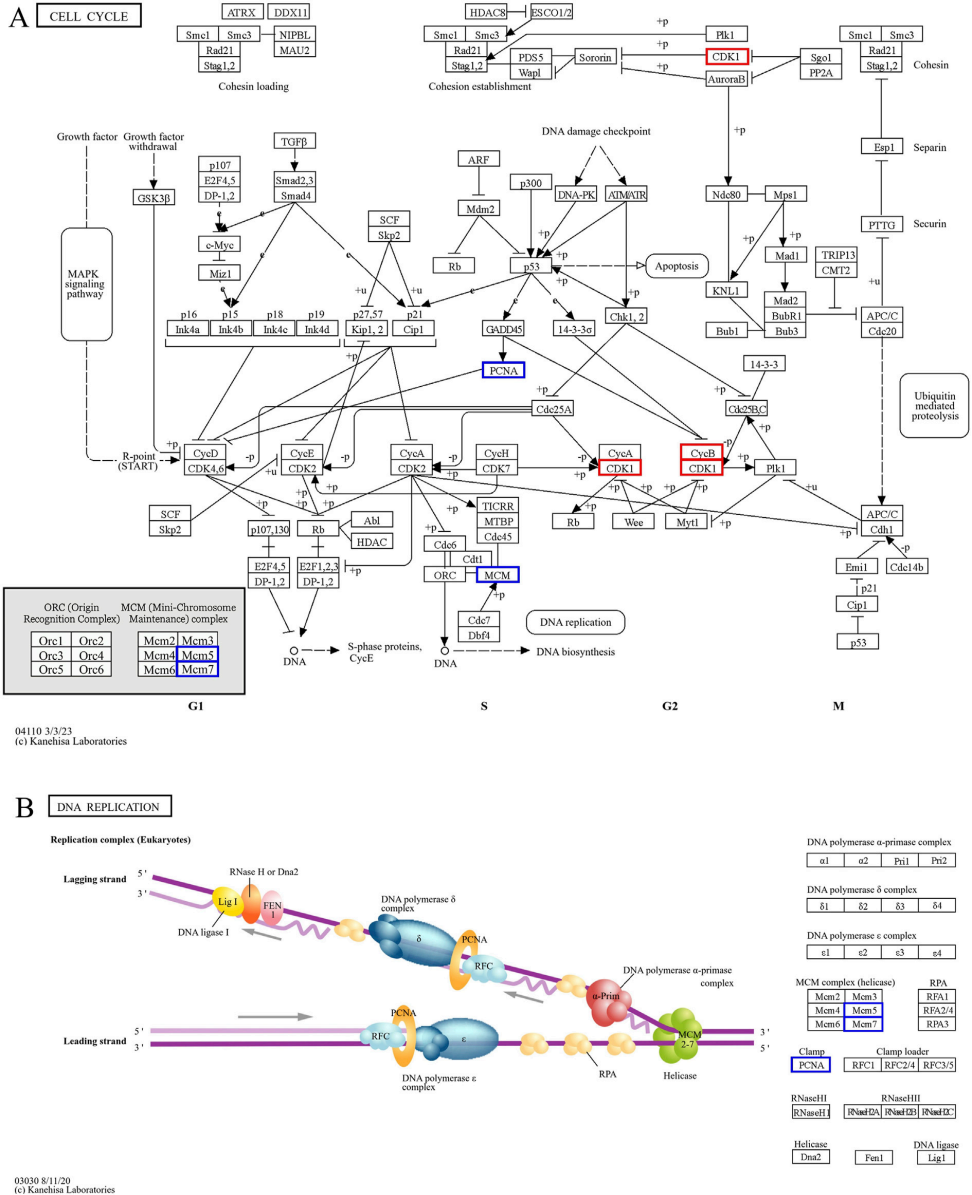
The hub genes related to **(A)** the cell cycle and **(B)** DNA replication KEGG pathway of downregulated DEGs in developmental groups, which contributed to arrested developmental groups at the 2-cell and 4-cell stage of transgenic Asian elephant-pig inter-order cloned embryos. Red means the hub genes found in both Arr2cell and Arr4cell. Blue means the hub genes found only in Arr4cell.

The intersecting core genes in the Dev2cell group included *MET*, *HGF*, *PIK3CB*, *FGFR3*, *FGFR2*, *FLT1*, *VEGFA*, *PTK2*, *RIPK1*, *IKBKG*, *RBL1*, *CCNE1*, *CCNE2*, *FOXO3*, and *FGF19* (Figure 7A). For the Dev4cell group, the core genes were *JUN*, *IKBKB*, *RIPK1*, *IKBKG*, *RBL2*, *MDM2*, *CDKN1A*, *CYLD*, *CCNE2*, *CDKN2B*, *DDIT3*, *CASP2*, *CCND2*, *FOSL2*, *VEGFA*, *KDR*, and *SQSTM1* (Figure 7B). KEGG enrichment analysis indicated that the core genes in both Dev2cell and Dev4cell were similarly involved in the PI3K-Akt and MAPK signaling pathways (Figures 7E, 8F). Notably, in the Dev4cell group, core genes, namely, *JUN*, *IKBKB*, *IKBKG*, *CYLD*, *SQSTM1*, and *FOSL2* were linked to osteoclast differentiation and C-type lectin receptor signaling (Supplementary Table S9). In Arr2cell embryos, key core genes included *NDUFC2*, *NDUFS3*, *NDUFAB1*, *SDHC*, *CDK1*, *CCNB1*, *NUP54*, *NUP43*, *NUP37*, *NDC1*, and *LOC126060805* (Figure 7C), with significant enrichment in the nucleocytoplasmic transport pathway and oxidative phosphorylation pathway (Figure 7G; Supplementary Table S9). The Arr4cell group displayed key hub genes involved in metabolic pathways, including the TCA cycle, carbon metabolism, pyruvate metabolism, and oxidative phosphorylation, as well as cell cycle and DNA replication (Figure 7H; Supplementary Table S8). These core genes were *CDK1*, *CCNB1*, *MCM5*, *MCM7*, *PCNA*, *SDHB*, *SDHC*, *SUCLG1*, *DLAT*, *ME2*, and *LOC126082754* (Figure 7D).

**FIGURE 9:**
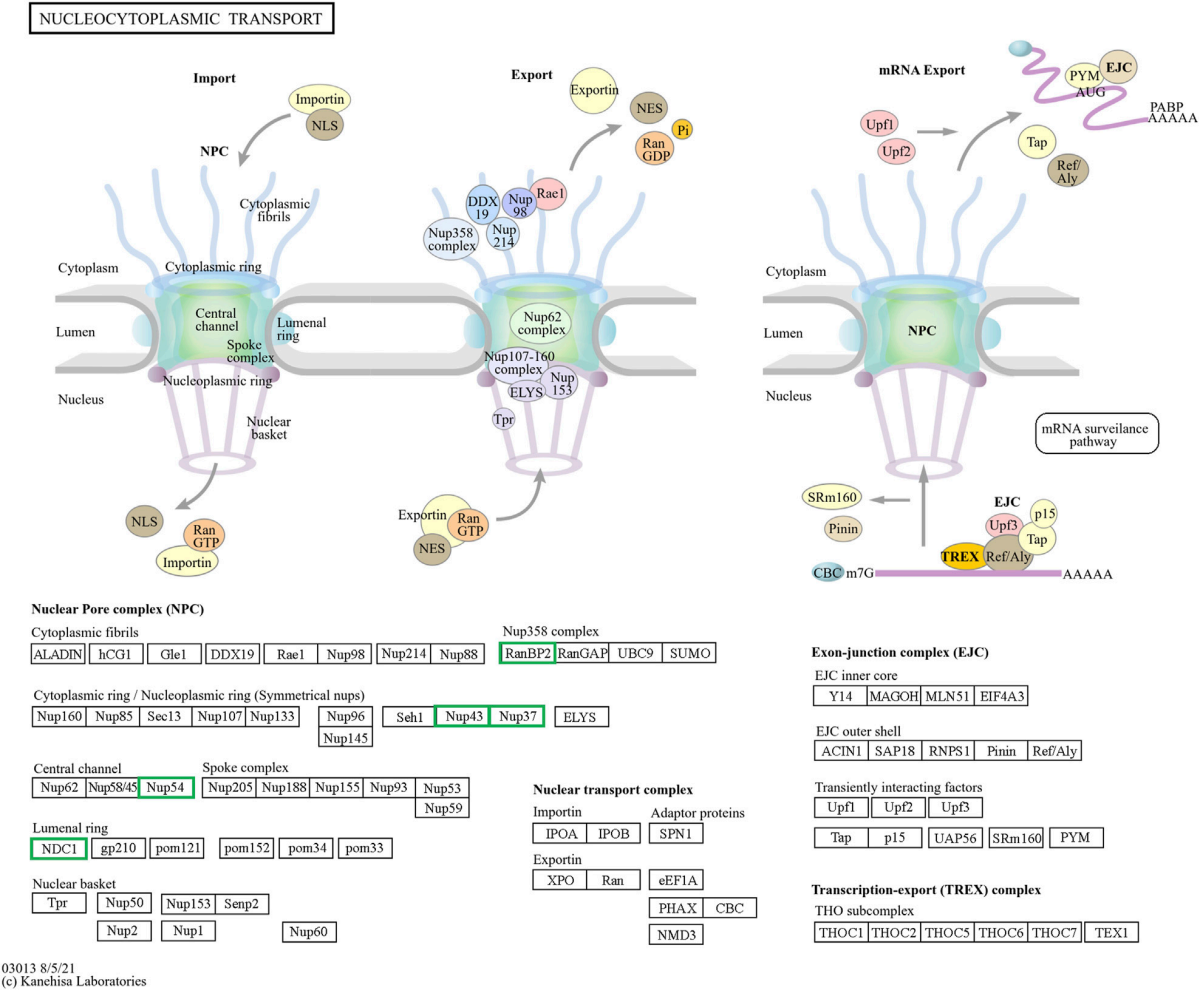
The hub genes related to the nucleocytoplasmic KEGG pathway of downregulated DEGs in developmental groups contribute to arrested developmental groups at the 2-cell stage of transgenic Asian elephant-pig inter-order cloned embryos. Green means the hub genes found only in Arr2cell.

**FIGURE 10:**
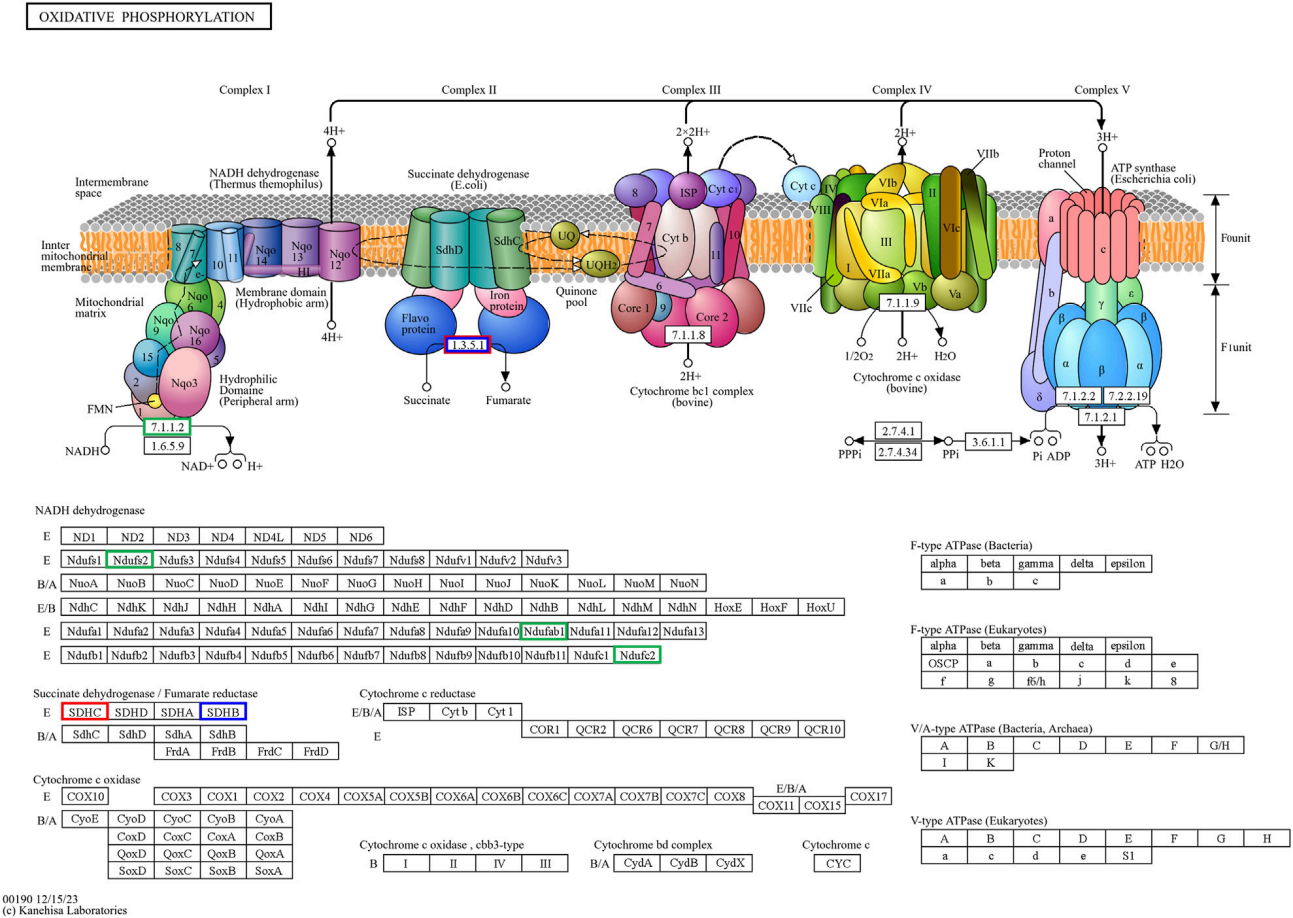
The hub genes related to the oxidative phosphorylation KEGG pathway of downregulated DEGs in developmental groups contribute to arrested developmental groups at the 2-cell stage of transgenic Asian elephant-pig inter-order cloned embryos. Green means the hub genes found only in Arr2cell. Blue means the hub genes found only in Arr4cell. Red means the hub genes found in both Arr2cell and Arr4cell.

The pathway enrichment analysis revealed that hub genes in the Arr2cell group were significantly associated with several critical pathways (Supplementary Table S9). Specifically, *CDK1* and *CCNB1* were associated with cell cycle progression (Figure 8). The key genes including *LOC126060805* (*RanBP2*), *NUP54*, *NUP43*, *NUP37*, and *NDC1* were related to nucleocytoplasmic transport pathways (Figure 9). Additionally, the core genes *NDUFC2*, *NDUFS3*, *NDUFAB1*, and *SDHC* were involved in energy metabolism pathways such as oxidative phosphorylation (Figure 10) and thermogenesis. For the Arr4cell group, the enrichment pathways analysis identified key hub genes associated with critical cellular pathways (Supplementary Table S9). Specifically, genes related to cell cycle regulation and DNA replication, namely, *CDK1*, *CCNB1*, *MCM5*, *MCM7*, and *PCNA* were significantly enriched (Figure 8). Moreover, pathways involved in energy production, such as the TCA cycle, carbon metabolism, pyruvate metabolism, and oxidative phosphorylation, were linked to core genes *SDHC*, *SDHB*, *SUCLG1*, *DLAT*, and *ME2* at the 4-cell stage in arrested transgenic AE-P iSCNT embryos (Figure 10).

## 4 Discussion

This study obtained the transcriptomic profiles of AE-P iSCNT embryos, by tagging Asian elephant donor cells with EGFP, facilitating the identification of embryos with the donor genome for RNA sequencing. Genome mapping analysis of AE-P inter-order cloned embryos showed approximately 25% of the genome and 15% of the genes matched the Asian elephant reference. These findings contribute a specific and valuable dataset on genome mapping in distant phylogenetic relationships, which could serve as a reference for other iSCNT species investigations.

Since elephant MZT/EGA remains unexplored, we observed these transitions in Asian elephant inter-order SCNT embryos through transcriptome profiling from the 2-cell to 8-cell stages. Our results demonstrated that transcriptional initiation and translation-related processes in AE-P iSCNT embryos likely reflect the onset of MZT/EGA during the D2D4 and D4D8 transition, respectively. EGA is typically delayed in SCNT embryos, likely due to aberrant epigenetic reprogramming (Niu et al., 2008; Cao et al., 2014; Wang et al., 2020). In porcine SCNT embryos, EGA is postponed to the 8-cell stage, compared to the 4-cell stage in IVV-derived counterparts (Cao et al., 2014). Similarly, delayed EGA has been observed in monkey SCNT embryos compared to IVF-derived embryos (Niu et al., 2008). Furthermore, challenges such as nDNA-mtDNA mismatch, nucleocytoplasmic incompatibility, and mtDNA heteroplasmy often disrupt EGA initiation in iSCNT embryos (Takeda, 2019; Mrowiec et al., 2021). For instance, bovine–pig iSCNT embryos failed to initiate EGA due to nucleocytoplasmic incompatibility, as evidenced by the absence of RNA Pol II accumulation, nucleoli formation, and developmental arrest (Lagutina et al., 2010). However, our RNA-seq data revealed partial transcriptional initiation and pathway enrichment in AE-P embryos, with 1,206 DEGs in D2D4 and 302 in D4D8, potentially pointing to a less robust EGA compared to those of IVV and SCNT porcine embryos (Zhai et al., 2022). Therefore, determining MZT/EGA in iSCNT embryos requires further investigation. Integrating genetic engineering technologies and multi-omics sequencing analyses across developmental stages could provide deeper insights into the mechanisms underpinning MZT/EGA in iSCNT.

In this study, we utilized porcine recipient oocytes, recognized as universal cytoplasts in iSCNT due to their strong demethylation capability, which enables nucleolar formation in donor nuclei from various species (Gupta et al., 2013; Lagutina et al., 2013). However, iSCNT developmental challenges increase with greater phylogenetic distance between donor and recipient species, exacerbating aberrant epigenetic reprogramming issues with residual somatic transcriptional memory and EGA asynchrony (Lagutina et al., 2013; Zhou et al., 2020; Adams et al., 2024). Our previous research demonstrated that AE-P iSCNT embryos could develop *in vitro* but failed to progress *in vivo* (Nguyen et al., 2022). This developmental limitation is likely attributable to the significant evolutionary divergence between Asian elephants (*E. maximus*) and pigs (*Sus scrofa*), estimated to span tens of millions of years based on molecular clock studies (Meredith et al., 2011). While closely related species can support iSCNT embryonic development (Gómez et al., 2004; Oh et al., 2008; Wani et al., 2017), distant species face nDNA-mtDNA incompatibilities causing developmental failure as early as the first cell division (Ikumi et al., 2004; Kato et al., 2009; Zuo et al., 2014; Son et al., 2021). This challenge underscores the limited reprogramming capacity of recipient cytoplasts from phylogenetically distant species, which struggle to effectively reprogram the donor nucleus.

We then analyzed the transcriptomic profiles and DEGs in AE-P iSCNT embryos, classifying their developmental progress at 24- and 48-h post-activation (the 2- and 4-cell stages, respectively). Arrested embryos were defined as those that ceased division for at least 24 h (Sfakianoudis et al., 2021); however, to exclude transiently arrested embryos (Montjean et al., 2021), we extended the observation period to 48 h apart. Our DEG analysis identified key developmental pathways in D2 and D4, including MAPK, Wnt, PI3K-Akt, Ras, and Rap1, which are known to be essential for embryonic development (Lee et al., 2014; Schulz and Harrison, 2019). Additionally, upregulated DEGs in these groups were enriched in pathways related to osteoclast differentiation and various immune processes. Key core genes, including *MET* and *HGF* in the D2 group, and *JUN*, *IKBKB*, *IKBKG*, and *FOSL2* in the D4 group, may reflect residual transcriptomic memory and premature gene expression, aligning with findings in previous studies (Ikeda et al., 2008; Desole et al., 2021; Su et al., 2023; Zhang J. et al., 2023). The AE-P iSCNT embryos in the D2 and D4 groups likely correspond to previous transcriptomic analyses of early-dividing SCNT embryos, which also retain somatic memory genes such as *FLRT2*, *ADAMTS1*, and *FOXR1* (Liu et al., 2020). These observations align with our findings and highlight the link between residual transcriptomic memory and incomplete reprogramming—likely influenced by epigenetic modifications crucial for erasing somatic cell memory during SCNT/iSCNT reprogramming (Wang et al., 2011; Long et al., 2014).

In our study, significant epigenetic DEGs including *DNMT1*, *KDM5B*, and *KDM4A* were identified in D2A2 and D4A4 embryos, indicating aberrant epigenetic reprogramming, which could impede somatic cell reprogramming and embryonic progression. Higher *DNMT1* expression during EGA was found to increase developmental arrest in porcine SCNT than in IVF embryos (Song et al., 2017). Moreover, inactivation of histone modification genes has stage-specific effects on cloned mouse embryo development; inactivating *KDM4B* reduces arrest at 2-cell, while the inactivation of *KDM5B* promotes 4-cell arrest (Liu et al., 2016). Although AE-P iSCNT embryos classified as normally developing followed the expected developmental timing observed in our previous study (Nguyen et al., 2022), this does not ensure developmental success, as some embryos might fail at later stages. To address these issues, we propose that future iSCNT studies, with limited established embryonic developmental references, should include more time points (e.g., 18-, 24-, 36-, and 48-h post-activation) and embryonic stages to further elucidate transcriptome dynamics and the MZT/EGA transition in iSCNT embryos. Integrating transcriptomic data with epigenetic analyses, such as chromatin immunoprecipitation followed by sequencing (ChIP-seq) (Liu et al., 2016), could provide more comprehensive insights into epigenetic modification events during iSCNT embryogenesis.

nDNA-mtDNA incompatibilities from donor and recipient genomes mismatches could disrupt gene function and cause developmental arrest. Here, we categorize the core genes of arrested embryos into three functional and pathway-related groups. First, genes related to the cell cycle and DNA replication, including *CDK1*, *CCNB1*, *MCM5*, *MCM7*, and *PCNA*, were identified. Since EGA success relies on precise cell cycle progression, disruptions in these genes could impair the G2/M transition, leading to G2 phase arrest, triggering stress, or apoptosis (Strauss et al., 2017; Alfonso-Pérez et al., 2019). Additionally, issues with spindle checkpoint assembly or DNA synthesis could also result in aneuploidy, genomic instability, and cell cycle arrest (Hayward et al., 2019; Kermi et al., 2019; Hu and Stillman, 2023). Upregulated DEGs in A2 and A4 identified GO enrichment terms such as mRNA splicing and cell cycle regulation, including the anaphase-promoting complex and negative regulation of G2/M transition. Therefore, these results suggest that arrested AE-P iSCNT embryos may face challenges in replication regulation, mitosis progression across the S, G2, and M phases. The next group of core genes such as *LOC126060805* (*RanBP2*), *NUP54*, *NUP43*, *NUP37*, and *NDC1* were related to nucleocytoplasmic transport pathway. *NUP*-related genes contribute to nuclear pore complex (NPC) formation by encoding nucleoporin (NUP) proteins, which facilitate efficient maternal transcription factor transport during MZT/EGA upon NPC maturation (Shen et al., 2022). For example, NUP37 and NUP43 form part of the scaffold outer ring, while NUP62-NUP58-NUP54 form the central transport channel, and NDC1 serves as a transmembrane component in humans (Chug et al., 2015; Schwartz, 2016). Studies have shown that NUP37 promotes nuclear import via the YAP1–TEAD pathway (Luo et al., 2017), whereas NUP54 supports genome integrity through homologous recombination repair under stress (Rodriguez-Berriguete et al., 2018). While NPCs share structural similarities across species, individual NUP compositions vary due to species-specific adaptations (Krull et al., 2004; Guglielmi et al., 2020; Schuller et al., 2021). Inefficiencies in NPC assembly and maturation probably hinder nuclear-cytoplasmic communication in arrested AE-P iSCNT embryos.

Finally, core genes in arrested embryos were linked to energy metabolism pathways, with upregulated DEGs enriched in nucleocytoplasmic transport and oxidative phosphorylation in A2, and other metabolic pathways like TCA cycle and pyruvate metabolism in A4. During cleavage stages in several mammalian species, glycolysis-derived pyruvate is metabolized through the TCA cycle and oxidative phosphorylation within the mitochondria to generate ATP (Zhao et al., 2023; Lee and Rinaudo, 2024). Early-stage cloned embryos rely on recipient oocyte mitochondria and *in vitro* culture nutrients to meet energy demands, as oxidative phosphorylation is the primary energy production in pre-implantation embryos (Zhang et al., 2007; Zuo et al., 2016; Cordova et al., 2017; Srirattana and St John, 2017). The development of bovine SCNT embryos from mtDNA-depleted donor cells to the blastocyst stage proceeds without disruption, highlighting the critical dependence on recipient oocyte mitochondria for early embryonic energy production in SCNT (Srirattana and St John, 2017). In arrested AE-P embryos, core genes such as *NDUFC2, NDUFS3, NDUFAB1, SDHC, SDHB, SUCLG1,* and *DLAT*, were enriched in oxidative phosphorylation, TCA cycle, carbon metabolism, and pyruvate metabolic pathways, which essential for embryonic growth, cell signaling, gene regulation, and differentiation (Zhao et al., 2023). Among these genes, *NDUFC2*, *NDUFS3*, and *NDUFAB1* are subunits of Complex I in the mitochondrial electron transport chain (ETC) (Hirst, 2013; Sazanov, 2015), while *SDHC* and *SDHB* belong to Complex II (Bezawork-Geleta et al., 2017). Disruptions in these genes may result in faulty, ETC., complex assembly, increased oxidative stress, and impaired ATP production, all of which may contribute to embryonic arrest (Hirst, 2013; Sazanov, 2015; Kwon et al., 2016; Zhao et al., 2019). Notably, the protein subunits of ETC Complex I are encoded by both nDNA and mtDNA, whereas those of Complex II are encoded solely by nDNA (St John et al., 2004; Bezawork-Geleta et al., 2017; Zhao et al., 2019). This inherent nDNA-mtDNA mismatch in iSCNT embryos likely hinders their, ETC., subunit assembly and energy production (Zuo et al., 2014; Kwon et al., 2016; Ma et al., 2016; Mrowiec et al., 2021). Altogether, these upregulated DEGs observed in arrested AE-P iSCNT embryos may represent compensatory responses, though their exact roles require further functional validation.

Conclusion, this study presents the first transcriptomic profiling of transgenic Asian elephant embryos produced through iSCNT using EGFP-expressing Asian elephant nuclei and porcine cytoplasts. The low mapping percentages of the AE-P iSCNT embryo genome and genes to the Asian elephant reference, approximately 25% and 15%, respectively, reflect the phylogenetic divergence between Asian elephants and pigs, potentially indicating the limited reprogramming efficiency of Asian elephant somatic nuclei using porcine cytoplasts. Our findings revealed that earlier-cleaving embryos at 2- and 4-cell stages exhibit incomplete epigenetic reprogramming, whereas arrested embryos display disruptions in cell cycle regulation, nucleocytoplasmic transport, and energy metabolism pathways. By examining transcriptomic profiles and key hub genes, this groundwork underscores the developmental barriers in inter-order AE-P iSCNT embryos, including challenges in nuclear and epigenetic reprogramming, nucleocytoplasmic incompatibility, and nDNA-mtDNA mismatches. These findings provide valuable insights into the molecular mechanisms, highlighting potential complexities that may need to be addressed in future studies to further advance the field of reproductive biotechnology and explore its potential applications in regenerative medicine and wildlife conservation.

## Data Availability

The data presented in the study are deposited in the National Center for Biotechnology Information (NCBI) repository, accession number PRJNA1186842.

## Supplementary material

SUPPLEMENTARY TABLE S1 List of elephant-specific TYR and GFP primers.

SUPPLEMENTARY TABLE S2 Quality control metrics of the clean reads per sample after data filtering.

SUPPLEMENTARY TABLE S3 Average clean reads per sample aligned to the Asian elephant genome and gene set.

SUPPLEMENTARY TABLE S4 GO terms from enrichment analysis with Q < 0.05 (top 20), considered significantly enriched by total DEGs from D2D4 and D4D8, as well as upregulated and downregulated DEGs from D2A2 and D4A4 groups.

SUPPLEMENTARY TABLE S5 KEGG pathways from enrichment analysis with Q < 0.05 (top 10), considered significantly enriched by total DEGs from D2D4 and D4D8, as well as upregulated and downregulated DEGs from D2A2 and D4A4 groups. Pathways with Q > 0.05 are highlighted in red.

SUPPLEMENTARY TABLE S6 Log2 (FPKM+1) values of DEGs used for heatmap clustering visualization, including A2D2D4, A4D4D8, and epigenetic-related DEGs in D2A2 and D4A4 groups.

SUPPLEMENTARY TABLE S7 The top 20 overlapping core genes identified as significant by PPI network analysis across MCC and Degree centrality algorithms. These genes, consistently scoring high for connectivity, were identified in upregulated and downregulated DEGs in D2A2 groups.

SUPPLEMENTARY TABLE S8 The top 20 overlapping core genes identified as significant by PPI network analysis across MCC and Degree centrality algorithms. These genes, consistently scoring high for connectivity, were identified in upregulated and downregulated DEGs in D4A4 groups.

SUPPLEMENTARY TABLE S9 The overlapping core genes, classified into groups based on relevant pathways, include core genes from upregulated and downregulated DEGs in D2A2 and D4A4 groups.
